# Gene-body DNA methylation of ONECUT2 predicts its expression and prostate cancer aggressiveness in needle biopsies

**DOI:** 10.1186/s40364-026-00890-7

**Published:** 2026-01-16

**Authors:** Yohei Sekino, Hong-Tao Li, Masatomo Kaneko, Yuta Inoue, Lorenzo Storino Ramacciotti, Rongying Lu, Zhenzhong Deng, Xinyi Zhou, Michelle Mingxue Song, Aditya Desai, Mingda Jin, Wei Guo, Xiaojing Yang, Jeffrey Bhasin, Nobuyuki Hinata, Michael R. Freeman, Inderbir Gill, Manju Aron, Steven Yong Cen, Andre Luis Abreu, Gangning Liang

**Affiliations:** 1 Department of Urology, USC Norris Comprehensive Cancer Center, University of Southern California, Los Angeles, CA 90089 USA; 2 Department of Urology, Hiroshima University Graduate School of Biomedical Sciences, Hiroshima, Japan; 3 Department of Urology, Kyoto Prefectural University of Medicine, Kyoto, Kamigyo-ku 602-8566 Japan; 4 Zymo Research Corp, Irvine, CA USA; 5 Departments of Urology and Biomedical Sciences, Samuel Oschin Comprehensive Cancer Institute, Cedars-Sinai Medical Center, Los Angeles, CA 90048 USA; 6 Department of Pathology, Norris Comprehensive Cancer Center, University of Southern California, Los Angeles, CA 90089 USA; 7 Department of Radiology/Neurology, University of Southern California, Los Angeles, CA 90089 USA; 8 Present Address: Institute of Brain Science and Brain-inspired Research, Shandong First Medical University & Shandong Academy of Medical Sciences, Jinan, 250117 China

**Keywords:** ONECUT2, Prostate cancer, Gene-body, DNA methylation, Gene expression, Aggressive prostate cancer, Needle biopsy, Biomarker, Therapeutic target

## Abstract

**Background:**

ONECUT2 is a lineage plasticity driver and therapeutic target in aggressive prostate cancer (PCa). This study investigated whether ONECUT2 gene-body DNA methylation regulates its expression and assessed its potential as a biomarker in clinical specimens.

**Methods:**

We analyzed associations between ONECUT2 gene-body methylation, expression, and patient survival across multiple datasets. The effect of DNA methylation on ONECUT2 expression was tested in prostate cancer cell lines using a DNA methyltransferase inhibitor (DNMTi). Validation was further performed in needle biopsy samples by targeted bisulfite sequencing for DNA methylation and RT-PCR for gene expression.

**Results:**

ONECUT2 expression strongly correlated with gene-body DNA methylation and patient survival in multiple datasets. DNMTi treatment confirmed this relationship in prostate cancer cells. In 208 biopsies from prostate cancer patients, hypermethylation of gene-body of ONECUT2 was linked to higher ONECUT2 expression and effectively distinguished tumor from adjacent normal tissue (*p* < 0.001 and AUC = 0.86). It also predicted aggressive features, including higher Gleason score (*p* = 0.01 and AUC = 0.68), advanced T stage (*p* = 0.04 and AUC = 0.65), seminal vesicle invasion (*p* = 0.0024 and AUC = 0.76), and lymph node involvement (*p* = 0.0005 and AUC = 0.80).

**Conclusion:**

Assessing ONECUT2 gene-body methylation in biopsies may serve as a surrogate for ONECUT2 expression and provide predictive insights into disease progression before surgery. Furthermore, suppressing ONECUT2 through DNMTi treatment represents a potential therapeutic strategy for aggressive PCa.

**Supplementary Information:**

The online version contains supplementary material available at 10.1186/s40364-026-00890-7.

## Background

Prostate cancer (PCa) is one of the most prevalent cancers globally [1] and is the leading cause of cancer-related death among men in a quarter of the world’s countries [2]. While localized PCa is often curable through radical prostatectomy or radiation therapy, metastatic PCa poses a life-threatening challenge. Despite the importance of early screening for PCa, current diagnostic and prognostic tools contribute to overdiagnosis and overtreatment [3]. Consequently, there is an urgent need for novel biomarkers to improve the accuracy of diagnosis and prognosis.

The transcription factor ONECUT2 serves as a master transcriptional regulator that suppresses the androgen axis and promotes lineage plasticity in PCa [4]. ONECUT2 is active in prostate adenocarcinoma as a lineage plasticity driver [5], but also promotes neuroendocrine prostate cancer (NEPC), a highly aggressive variant that emerges as a result of AR-targeted therapy [6] and ONECUT2 is active during hypoxia signaling [4, 7]. ONECUT2 can be directly targeted with a novel small molecule, CSRM617, that was demonstrated to suppress tumor growth and metastasis in vivo [4]. These findings underscore the crucial role of ONECUT2 in PCa development and progression. Understanding the mechanisms underlying ONECUT2 regulation holds promise in the assessment of therapeutic strategies for PCa patients.

DNA methylation is an epigenetic mechanism that plays a crucial role in various biological processes in cancer [8, 9]. Given the cancer-specific nature of DNA methylation alterations [8, 10] and their general stability, DNA methylation aberrancies can serve as biomarkers for monitoring cancer severity and prognosis [9, 11]. Global DNA methylation analyses have revealed its widespread presence, not only in gene promoters but also in gene bodies. Typically, DNA methylation in the gene promoter region is associated with gene silencing [12], but conversely, our group and others have demonstrated that gene-body DNA methylation can be positively correlated with gene expression, thereby enhancing transcription activity [13, 14]. Although we have identified DNA methylation markers to predict the aggressive prostate cancer [15], the epigenetic regulatory role of the gene-body methylation in PCa remains incompletely elucidated. Therefore, we aim to analyze the potential role of ONECUT2 gene-body methylation in its regulation and investigate its use as a PCa biomarker and/or therapeutic target.

Here, we demonstrated an increase in both transcriptional expression and levels of ONECUT2 gene-body DNA methylation in aggressive features of PCa using data from public databases. A strong positive correlation was observed between the level of ONECUT2 expression and DNA methylation of its gene-body. Moreover, gene-body DNA methylation was shown to regulate ONECUT2 expression in vitro. Additionally, targeted bisulfite sequencing of primary prostate tumor needle biopsy samples revealed an increase in ONECUT2 gene-body DNA methylation coincident with aggressive PCa features. This study highlights the involvement of gene-body DNA methylation in regulating ONECUT2 expression, emphasizing that ONECUT2 gene-body DNA methylation can serve as a novel biomarker including needle biopsy samples and/or therapeutic target for aggressive PCa.

## Methods

### Patients

#### DNA methylation and expression profiles from public datasets

The UCSC Xena web tool was used to determine ONECUT2 expression and DNA methylation data in The Cancer Genome Atlas (TCGA) for prostate adenocarcinoma (PRAD) dataset [16]. The expression array data were downloaded from Gene Expression Omnibus (GEO) under accession numbers, GSE3325 [17], GSE6919 [18], GSE21034 [19], GSE32269 [20], GSE32967 [21] GSE35988 [22], GSE66187 [23], GSE70770 [24], GSE77930 [25], GSE94767 [26], GSE104786 [27], and GSE126078 [28]. The NEPC dataset was downloaded from the study by Beltran et al. [29]. The DNA methylation data were downloaded from GEO and Array Express under accession numbers, E-MTAB-6131 [30], GSE73549 [15], GSE83917 [31], GSE107298 [32], GSE127985 [33], GSE157272 [34], GSE174613 [35], GSE183019 [36].

#### Patients with needle biopsies

PCa patients undergoing radical prostatectomy (RP) from April 2019 to July 2022 were prospectively recruited (IRB# HS-16-00404) and patient characteristics were summarized (Table 1**)**. Three-core biopsy (Ex-vivo) fresh tissues were precisely sampled from the same location in the right and left lobes of the ex vivo RP specimens (a total of six cores per patient). Then, one-core was histopathologically (H&E) analyzed by a pathologist and two cores were analyzed for DNA methylation (Supplementary Fig. 1A). We collected 393 cores in total, but 185 cores were excluded due to presurgical treatments such as deprivation hormone therapy or duplication or poor quality of DNA. Among 208 cores (112 patients), 140 cores were confirmed as adjacent normal (benign or pre-malignant) prostate, and 68 cores were confirmed as prostate cancer (Supplementary Fig. 1B).

**Table 1:**
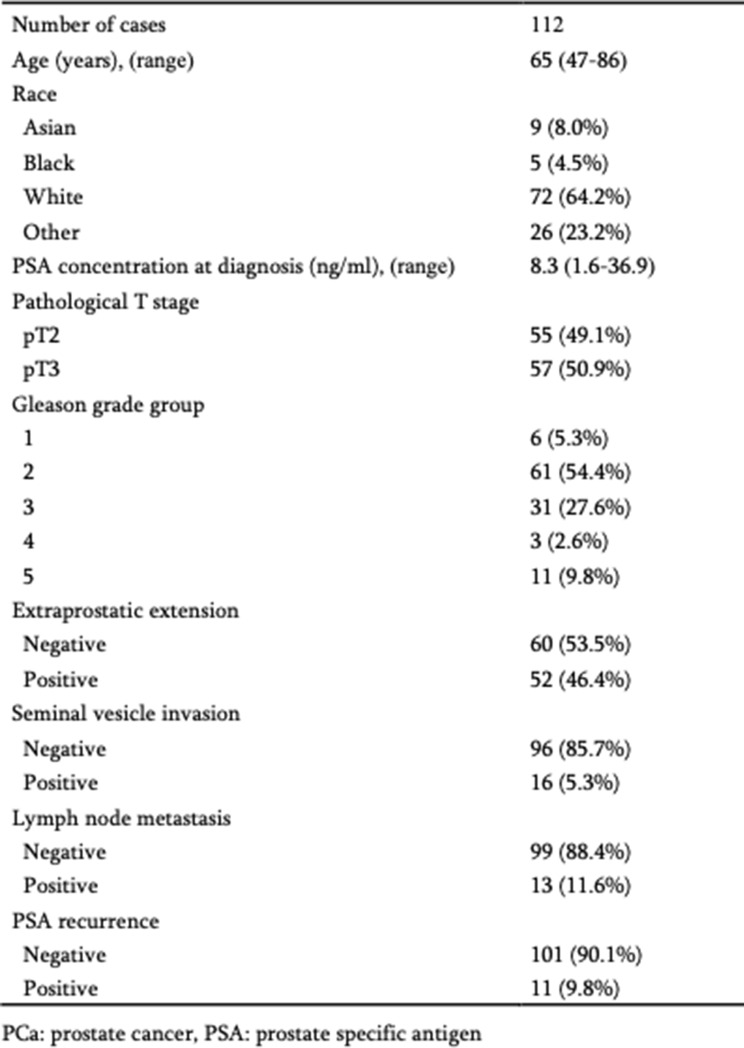
Clinicopathologic characteristics of 112 PCa patients

### Cell culture and drug treatments

RWPE1 (a non-tumorigenic parental prostate line originated from normal human prostate epithelium), C4-2B, PC3 and LNCaP (prostate cancer cell lines) were obtained from the American Type Culture Collection (ATCC; www.atcc.org). RWPE1 (CRL-3607™) cells were cultured in keratinocyte serum-free medium containing 50 µg/ml bovine pituitary extract and 5 ng/ml epidermal growth factor (Gibco-Thermo Fisher Scientific). C4-2B (CRL-3315™), LNCaP (CRL-1740™) and PC3 (CRL-1435™) cells were cultured in DMEM + L-Glutamine (Corning) and 10% Fetal Bovine Serum (Corning) and 1% penicillin-streptomycin (pen-strep, Genesee Scientific). 5-Aza-CdR/DAC (MedChem Express, Cat.: HY-A0004) was prepared at 100 µM in DMSO and was diluted in PBS to indicated concentrations before use.

### Western blotting

Total cell protein extracts were prepared by cell lysis with RIPA buffer (Thermo Scientific™, Cat.: 89900) supplied with Protease and Phosphatase Inhibitor (Thermo Scientific™ Halt™, Cat.: 78442). Total cell protein was mixed with NuPAGE™ LDS Sample Buffer (4X, Thermo Scientific™, Cat.: NP0007) and NuPAGE™ Sample Reducing Agent (10X, Thermo Scientific™, Cat.: NP0009) and then incubated at 98℃ for 5 min. Equal amounts of protein extracts were resolved by 4–15% precast polyacrylamide gel electrophoresis (BIO-RAD Mini-PROTEAN^®^ TGX™) and transferred to nitrocellulose membranes (BIO-RAD, #1704270). Immunoblotting was performed with primary antibodies against the following proteins: ONECUT2 (Proteintech, #21916-1-AP) and β-actin (Sigma-Aldrich, #A2228) and secondary antibodies: Goat anti Rabbit IgG (Fc): HRP (BIO-RAD, STAR121P), m-IgGκ BP-HRP (Santa Cruz, sc-516102).

### RNA Extraction, cDNA synthesis and quantitative RT-PCR assay for cell lines and patients’ samples

Total RNA from RWPE1, PC3 and C4-2B cells was extracted using the Qiagen RNeasy Mini Kit (Qiagen, Cat.:74106) followed by on-column DNase I digestion according to the manufacturer’s protocol at the indicated time after treatment. Total RNA (2 µg) was converted to cDNA with Moloney Murine Leukemia Virus Reverse Transcriptase (Promega). qRT-PCR was performed in CFX96 Touch Real-Time PCR Detection System (Bio-Rad) and CFX Opus 96 Real-Time PCR instrument using KAPA SYBR FAST qPCR Master Mix with ONECUT2 primers (Supplementary Table 1).

Total RNA from the biopsy specimen was extracted from the patient cores using the Quick-DNA/RNA™ Miniprep Plus Kit (Zymo Research) according to the manufacturer’s instructions. A total RNA (1 µg) was converted to cDNA using SuperScript III Reverse Transcriptase (Invitrogen). Multiplex qRT-PCR was performed in the CFX96 Touch Real-Time PCR Detection System (Bio-Rad) and the CFX Opus 96 Real-Time PCR instrument using PrimeTime^®^ Gene Expression Master Mix (IDT) with ONECUT2/HPRT1 primers and probes (Supplementary Table 1). RT-qPCR was performed for 40 cycles, and all samples were duplicated. The raw Cq values were exported and subsequently analyzed in R. A cutoff of 35 was established for the HPRT1 Cq values, and values above this threshold were deemed unsuitable and excluded from further analysis. For ONECUT2 and the reference gene HPRT1, a standard curve was generated using a PC3 serial dilution. The curve was fitted using linear regression of Cq versus log_10_ (input). PCR efficiency was calculated as E = (10^− 1/slope^ – 1) x 100%. The median slopes for HPRT1 and ONECUT2 were − 3.30 (interquartile range [IQR]: -3.45 to -3.20) and − 3.30 (IQR: -3.36 to -3.20), respectively. Absolute quantities for unknown samples were calculated per target as Q = 10^(Cq−intercept)/slope^. Target quantities for each sample were normalized to HPRT1 (Q_target_/Q_HPRT1_), and technical replicates were summarized as the mean +/- SD. Finally, the normalized values were scaled to a calibrator (LNCaP), set to 1, yielding relative expression. LNCaP was used only as a calibrator and was not used to be compared with samples. No-template and no-RT controls were run in parallel and excluded from quantification (all of the no-template and no-RT controls exhibited no amplification).

### DNA extraction and modified combined bisulfite restriction analysis (mCOBRA)

Genomic DNAs from RWPE1, PC3, and C4-2B cells after indicated treatments were purified using phenol–chloroform extraction and ethanol precipitation. A total of 500 ng DNA was bisulfite converted using the EZ DNA Methylation-Lightning Kit (Zymo research). The amplicons for bisulfite-treated DNA covering specific ONECUT2 CpG sites (cg10835584, cg24771804) was amplified by PCR (Supplementary Tables 1, 2). Bisulfite-PCR amplicons corresponding to each CpG site (cg10835584, cg24771804) were digested with the restriction enzyme BstUI (cut at CG/CG) and BsiWI (cut at C/GTACG) (NEB), respectively. The digested amplicons were amplified by qPCR using the same primers to test restriction enzyme digestion rates that can reflect the DNA methylation levels of each targeted CpG site. The relative DNA methylation levels were calculated by comparing the digestion rates between samples before and after drug treatments.

### DNA methylation analysis in needle biopsies

Tissue samples were immediately preserved in DNA/RNA Shield (Zymo Research) and genomic DNAs were purified using the Quick-DNA/RNA™ Miniprep Plus Kit (Zymo Research) according to manufacturer’s instructions. Sample library preparation and data analyses were performed by Zymo Research. Briefly, 200 ng genomic DNA from each sample was bisulfite-converted using EZ DNA Methylation-Lightning Kit (Zymo Research). Bisulfite-converted DNAs for the SWARM (Simplified Whole-panel Amplification Reaction Method) were prepared according to the manufacturer’s instructions. The ONECUT2 locus was amplified in chr18:55107668–55107793. The targeted primers were designed to cover two interrogated CpG sites (cg10835584 and cg24771804) but included a total of 8 CpG sites which was also analyzed in target sequencing (Forward: 5’-GGT TTT TTT TGG GTT TTY GGG GT-3’, Reverse: 5’-AAA ACC AAA TAC TTA CRC TAA AAA CTC TAC-3’). The letters R and Y denote standard IUPAC degenerate bases used in primer design for bisulfite-converted DNA. Specifically, Y indicates a C/T position (pyrimidine), and R indicates a G/A position (purine) where necessary to span unavoidable CpG positions in the primer binding regions. A mean of 1000x coverage was sequenced and read-level DNA methylation patterns of this amplicon were characterized by a bioinformatics workflow eliminating undesired noise derived from sequencing byproducts such as false CpG calls, dimers, and off-target alignments. The sequencing data is available at the Harvard Dataverse (https://dataverse.harvard.edu/dataset.xhtml? persistentId=doi:10.7910/DVN/UKER1G).

#### Statistical analyses

All experiments were repeated at least three times with each sample analyzed in triplicate. The results are expressed as the mean ± SD of the triplicate measurements. Statistical differences were evaluated using Mann-Whitney U-test. The one-way analysis of variance (ANOVA) is used to determine whether there are global differences in ONECUT2 expression level across the 5 Gleason categories, as an indicator of association between ONECUT2 expression and Gleason score [37]. After Kaplan-Meier analyses, any statistical difference between the survival curves of the cohorts was determined with the log-rank Mantel-Cox test. A p-value of < 0.05 was considered statistically significant. Statistical analyses were conducted primarily using GraphPad Prism software (GraphPad Software Inc., La Jolla), JMP17 (SAS Institute, Cary) or R version 4.4.2.

## Results

### The expression level of ONECUT2 is positively associated with PCa aggressiveness and poor patient outcomes

**Fig. 1:**
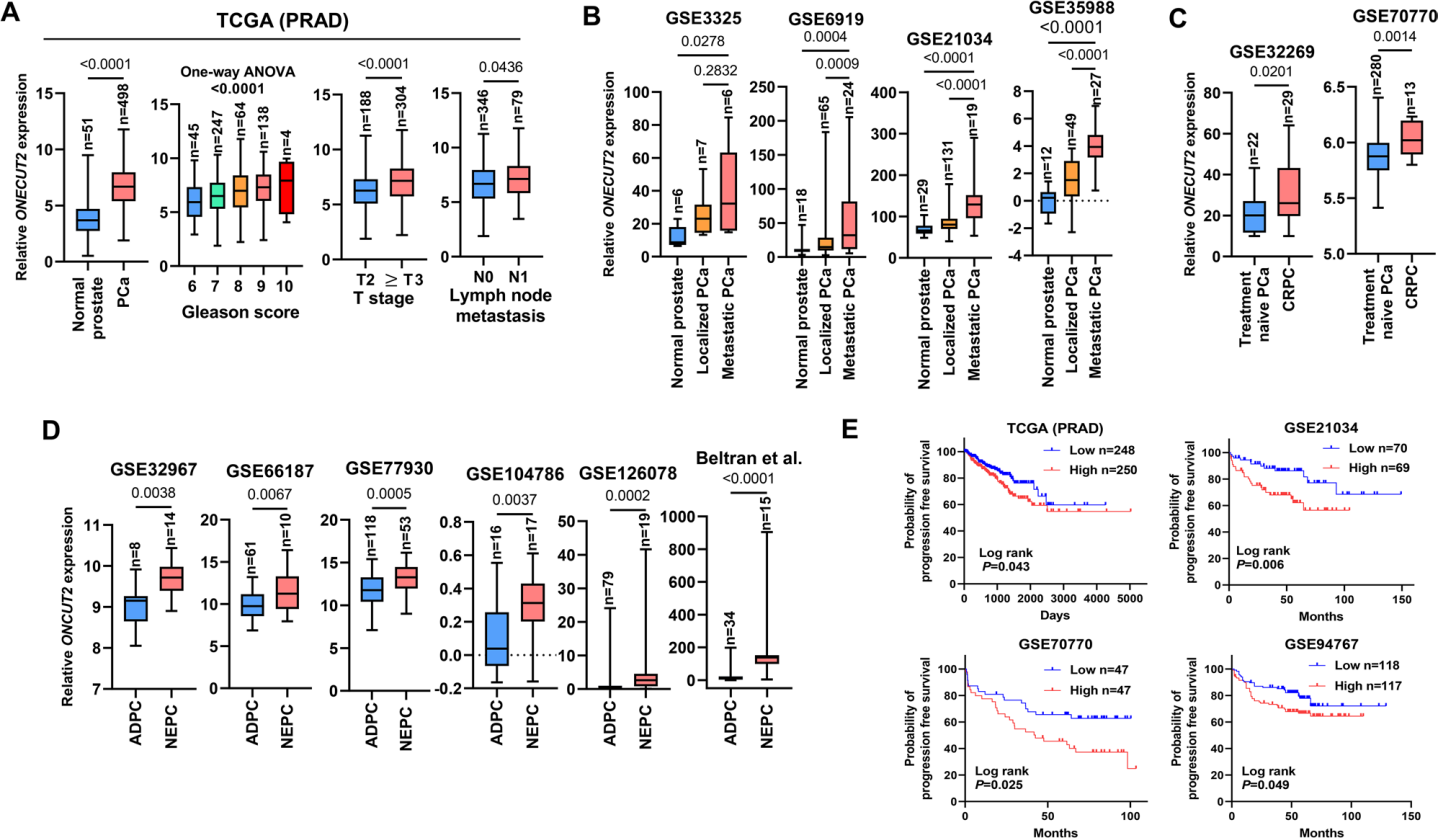
ONECUT2 expression in multiple public PCa datasets. (**A**) ONECUT2 expression in normal prostates and localized PCas from the TCGA PRAD dataset. (**B**) ONECUT2 expression in normal prostate, localized PCa and metastatic PCa from GSE3325, GSE6919, GSE21034, GSE35988 datasets. (**C**) ONECUT2 expression in treatment naïve PCa and castration resistant prostate cancer (CRPC) from GSE32269 and GSE70770 datasets. (**D**) ONECUT2 expression in adenocarcinoma prostate cancer (ADPC) and neuroendocrine prostate cancer (NEPC) from GSE32967, GSE66187, GSE77930, GSE104786, GSE126078 datasets and by Beltran et al. [29] (**E**) Kaplan-Meier plots of PSA recurrence-free survival of PCa patients stratified by ONECUT2 expression after prostatectomy using TCGA PRAD, GSE21034, GSE70770, and GSE94767 datasets

To characterize ONECUT2 transcript expression in PCa, we interrogated several public databases, including TCGA PRAD (prostate adenocarcinoma) and other published (GSE) datasets. Analysis of TCGA PRAD expression data revealed a significant increase in ONECUT2 expression in PCa, significantly associating with cancer presence, high Gleason score, high tumor stage, and positive lymph nodes (metastasis) (Fig. 1A). In examining additional multiple datasets (GSE3325, GSE6919, GSE21034, GSE35988), ONECUT2 expression was significantly increase in metastatic PCa compared to normal prostate and localized PCa (Fig. 1B). As anticipated, ONECUT2 expression was also significantly higher in castration-resistant PCa (CRPC) compared to treatment-naïve PCa (GSE32269 and GSE70770) (Fig. 1C). Furthermore, ONECUT2 expression was markedly increased in NEPC compared to prostate adenocarcinoma based on data retrieved from public databases (GSE66187, GSE77930, GSE104786, GSE126078, and Beltran et al. [29]). (Fig. 1D). Survival analyses of TCGA (PRAD), GSE21034, GSE70770, and GSE94767 data sets revealed that PCas with high ONECUT2 expression were strongly associated with poor recurrence-free survival (Fig. 1E). Taken together, these findings indicate that up-regulation of ONECUT2 expression is involved in biological oncogenic processes of PCa, particularly associating with disease aggressiveness.

### ONECUT2 gene-body DNA methylation is positively correlated with PCa aggressiveness and poor patient survival

**Fig. 2:**
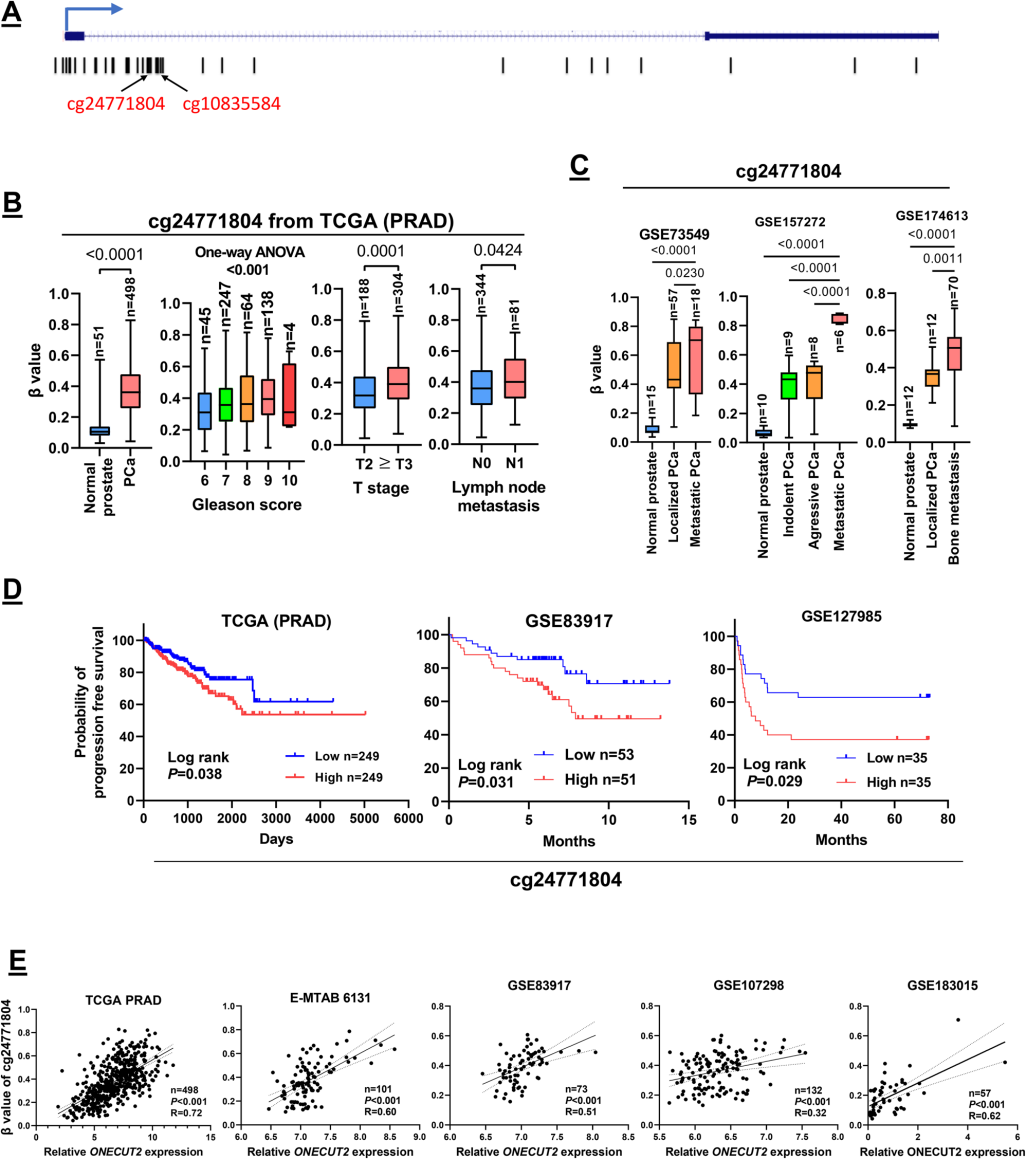
The DNA methylation status of cg24771804 located in the ONECUT2 gene-body in PCa. (**A**) Map of the ONECUT2 gene including promoter and transcribed regions. The tick marks represent CpG sites covered by the Illumina EPICv1 array. (**B**) The beta value of cg24771804 from TCGA PRAD tumors and normal-adjacent prostate tissues. (**C**) The beta value of cg24771804 in normal prostate, localized PCa, and metastatic PCa from GSE73549, GSE157272, and GSE174613 datasets. (**D**) Kaplan-Meier plots of PSA recurrence-free survival of PCa patients with the beta value of cg24771804 after prostatectomy from TCGA PRAD, GSE83917, and GSE127985 datasets. (**E**) The correlation between ONECUT2 expression and cg24771804 DNA methylation in the ONECUT2 gene-body based on the TCGA PRAD, E-MTAB 6131, GSE83917, GSE107298, and GSE183015 datasets. Correlation coefficients of each cohort are plotted

As mentioned above, DNA methylation levels may correlate with gene expression, with the relationship depending on the location of methyl-mark placement. In general, promoter DNA methylation is negatively correlated with gene expression at the transcriptional level, while gene-body DNA methylation is positively correlated with gene expression [9, 13]. A detailed diagram of the ONECUT2 gene is provided in Fig. 2A and includes both the promoter and transcribed regions (gene body), along with CpG sites measured by the Illumina Infinium DNA methylation BeadArray platform. Minimal promoter *ONECUT2* DNA methylation was observed in both PCa specimens and adjacent normal prostate tissues (TCGA PRAD) (Supplementary Fig. 2A). Among the CpG sites at ONECUT2 gene body (Supplementary Table 2), we selected two CpG sites (cg10835584 and cg24771804) for further analysis. The DNA methylation levels of these two CpG sites were significantly higher in localized PCa compared with normal prostate tissues and were also significantly elevated in cases with high Gleason scores, advanced T stage, and lymph node metastases (TCGA PRAD) (Fig. 2B and Supplemental Fig. 3A). Additionally, the DNA methylation at these two CpG sites was significantly increased in metastatic PCa across the GSE73549, GSE157272, and GSE174613 datasets (Fig. 2C and Supplemental Fig. 3B). The survival analysis revealed that high DNA methylation levels of these two CpG sites were associated with poor PSA recurrence-free survival in TCGA PRAD, GSE83917, and GSE127985 cohorts (Fig. 2D and Supplemental Fig. 3C). Furthermore, a positive correlation was observed between ONECUT2 expression and gene-body DNA methylation, particularly at two CpG sites, in TCGA PRAD as well as in several public datasets (E-MTAB6131, GSE107298, GSE183019) (Fig. 2B and Supplemental Fig. 3D). These results suggest a strong positive correlation between the gene-body DNA methylation and expression of ONECUT2 (Fig. 1) and proposes that ONECUT2 gene-body DNA methylation may serve as a promising biomarker for PCa progression and patient prognosis, not only representing increased DNA methylation, but also indicating the potential upregulation of the gene.

### ONECUT2 expression shows a strong positive association with gene-body DNA methylation in prostate cancer cell lines

**Fig. 3:**
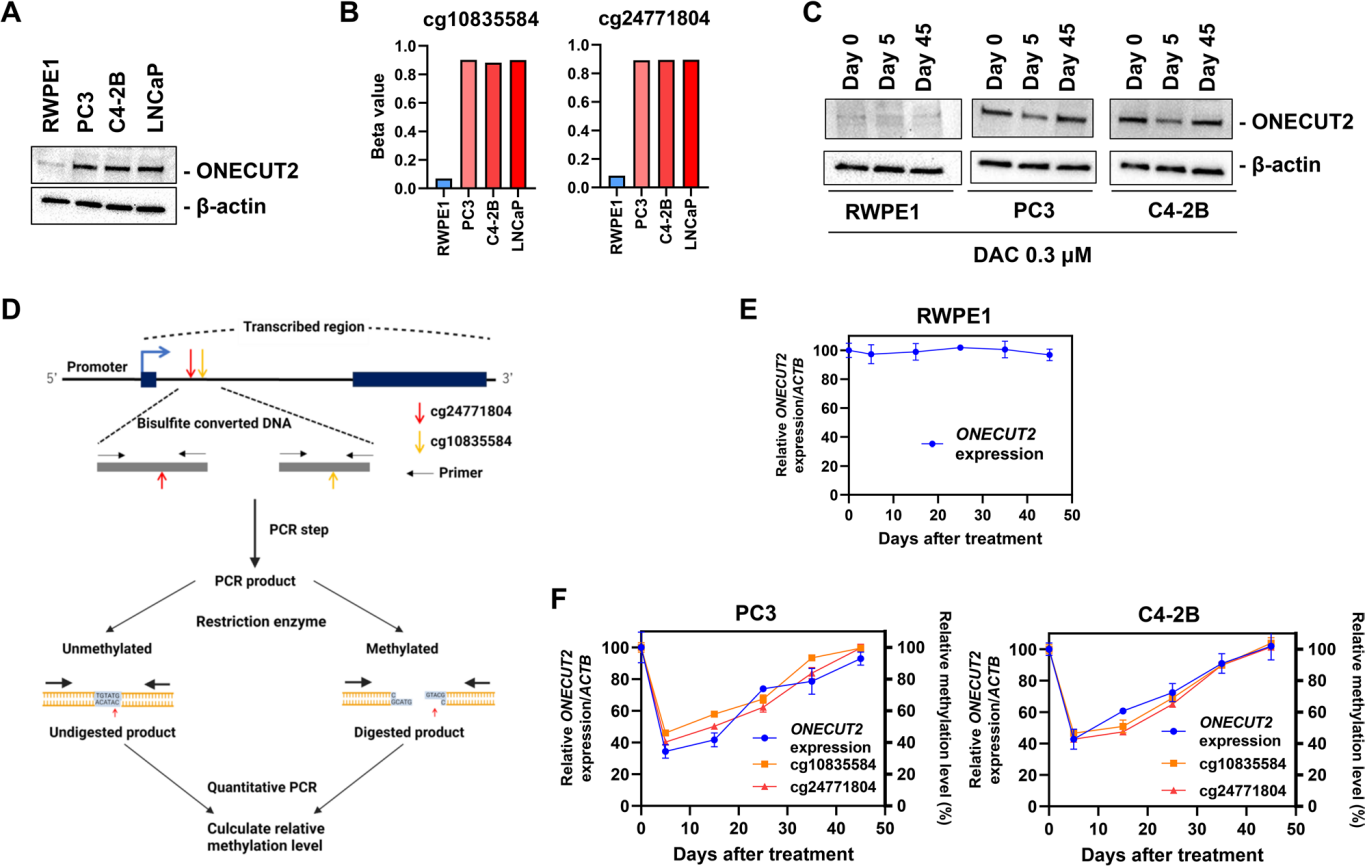
ONECUT2 expression shows a strong positive association with gene-body DNA methylation in prostate cancer cell lines. (**A**) Western blots of expression of ONECUT2 protein in RWPE1, PC3, C4-2B and LNCaP cells. β-actin was used as a loading control. (**B**) The beta value of two CpG sites (cg10835584, cg24771804) in the ONECUT2 gene-body in RWPE1, PC3, C4-2B and LNCaP cells. (**C**) The effect of DAC treatment on ONECUT2 protein expression level in RWPE1, PC3, and C4-2B cells. β-actin was used as a loading control. (**D**) Outline of the modified COBRA procedure. Figure was created using BioRender.com. (**E**) The effect of DAC treatment on the transcriptional expression of ONECUT2 in RWPE1 cells. (**F**) The effect of DAC treatment on the transcriptional expression of ONECUT2 and the DNA methylation levels of cg10835584 and cg24771804 in C4-2B and PC3 cells

Given the strong correlation between ONECUT2 expression and gene-body DNA methylation, we sought to investigate whether ONECUT2 gene-body DNA methylation alterations directly regulate its expression in human PCa cell lines. Western blot analyses revealed elevated ONECUT2 expression in PCa cell lines (PC3, C4-2B, LNCaP) compared to the normal prostate cell line (RWPE1) (Fig. 3A), with the promoter region remaining unmethylated in all four cell lines (Supplemental Fig. 2B). As anticipated, the two specific CpG sites within the gene-body (cg10835584 and cg24771804) were highly methylated in PCa cell lines but not in RWPE1 non-tumorigenic prostate cells (Fig. 3B), and ONECUT2 gene-body DNA methylation was highly positively correlated with the protein levels (Fig. 3A).

We next examined the effect of the demethylating agent decitabine (DAC) on ONECUT2 expression in human PCa cell lines. After treatment with 300 μm DAC for 24 h, ONECUT2 protein expression status was monitored at day 5 and 45 and compared with the expression before treatment (day 0). Western blotting showed that the low ONECUT2 protein expression in RWPE1 cells remained low before and after treatment at day 45 (Fig. 3C). In contrast, the highly expressed ONECUT2 protein levels were downregulated at day 5 after DAC treatment in PC3 and C4-2B, both of which have high basal gene-body DNA methylation (Fig. 3C). ONECUT2 expression recovered at day 45 after DAC treatment (Fig. 3C).

To directly monitor the correlation between ONECUT2 gene-body DNA methylation and mRNA expression, we analyzed the relationship between the gene-body methylation level of two CpG sites (cg10835584, cg24771804) and ONECUT2 transcript levels using the modified COBRA assay that combines the COBRA assay for DNA methylation (Fig. 3D) and quantitative PCR for expression. The COBRA assay is designed to determine DNA methylation levels at specific gene loci in small amounts of genomic DNA, in which restriction enzyme digestion is used to reveal methylation-dependent sequence differences in PCR products of sodium bisulfite converted DNA (Fig. 3D) [38]. The quantitative PCR for expression showed that there was no significant change in ONECUT2 expression in the non-tumorigenic RWPE1 prostate cell line (Fig. 3E), in which ONECUT2 gene-body was not methylated or minimal (Fig. 3B). Conversely, in PCa cell lines with extensive basal ONECUT2 gene-body DNA methylation (PC3, C4-2B) (Fig. 3B), both DNA methylation and ONECUT2 expression decreased by day 5 after DAC treatment. Interestingly, ONECUT2 gene-body DNA methylation and expression were finally restored to their original levels by day 45 after DAC treatment (Fig. 3F) and indicate that changes in ONECUT2 gene-body DNA methylation directly led to alterations in its expression. In other words, ONECUT2 expression may be directly regulated by gene-body methylation in PCa. This finding suggests that gene-body DNA methylation could serve as a therapeutic target for downregulation of ONECUT2 and/or blocking the ONECUT2 signaling pathway.

### Validation of ONECUT2 gene-body DNA methylation in PCa needle biopsies

Having demonstrated the association between ONECUT2 gene-body DNA methylation and expression with PCa progression and prognosis (Figs. 1 and 2), we aimed to leverage this novel discovery for clinical applications. Since prostate needle biopsies are crucial for PCa diagnosis, we evaluated our ONECUT2 gene-body DNA methylation marker on needle biopsy samples, guided and mapped in 3D by fused ultrasound and MR images. By combining image-guided targeted biopsy with our DNA methylation markers, we aimed to enhance the detection of all foci and assess their aggressiveness, particularly focusing on the role of ONECUT2 DNA methylation in mCRPC and its potential as a therapeutic target. This approach holds promise for guiding decisions on focal ablation therapy.

**Fig. 4:**
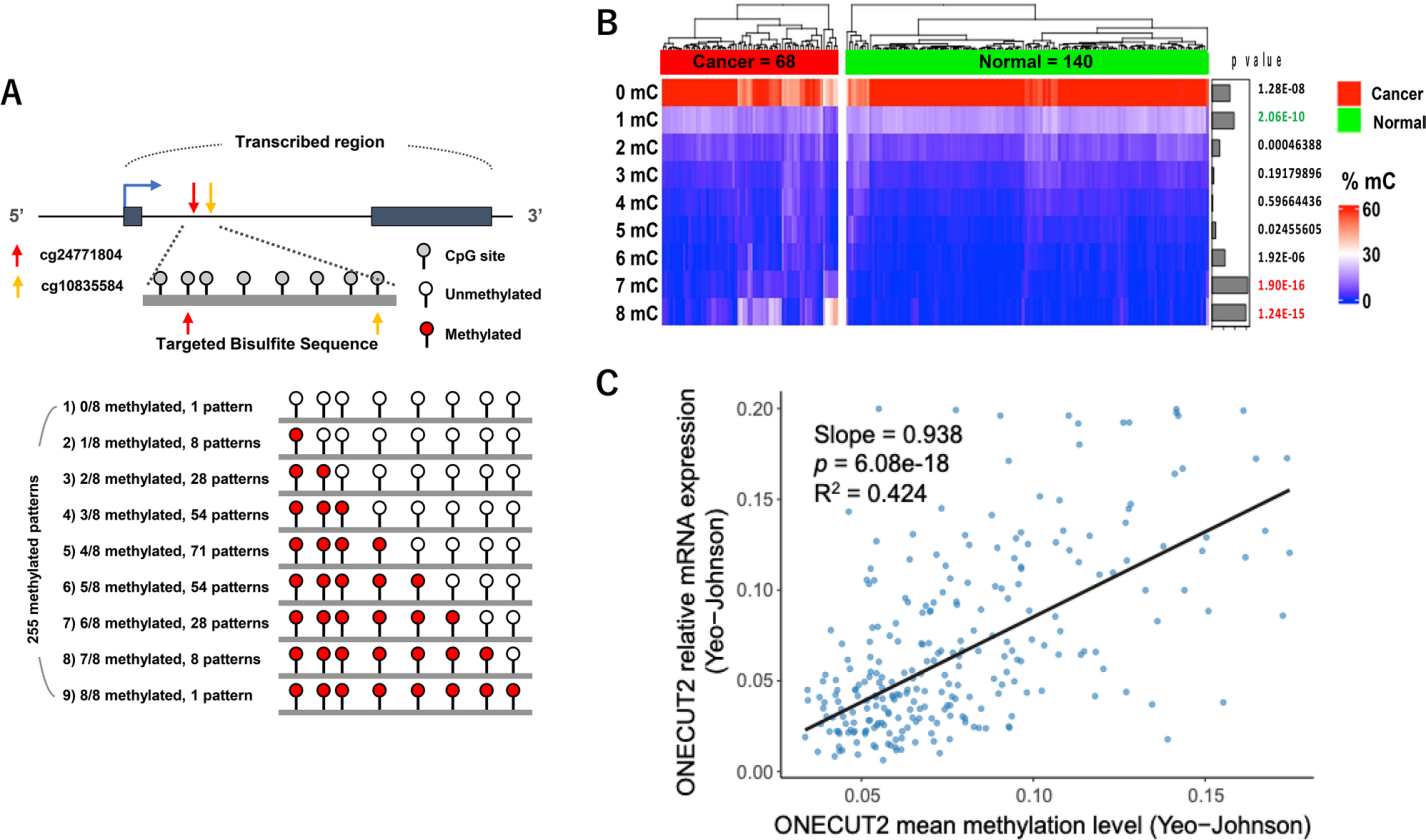
ONECUT2 gene-body DNA methylation based on targeted bisulfite sequencing and correlation with gene expression in needle biopsies. (**A**) The experimental outline of the targeted bisulfite sequencing approach. Figure was created using BioRender.com. (**B**) Heatmap of the ONECUT2 gene-body DNA methylation pattern based on DNA methylation status of 0–8 CpGs in the gene-body region. 7–8 CpGs P value labed as red represented most signifcant difference between normal and cancer. (**C**) Robust linear regression analysis comparing the relationship between ONECUT2 gene-body DNA mean methylation levels and mRNA relative expression compared to LNCaP in needle biopsies. These data were converted from raw data using a Yeo-Johnson transformation

We prospectively collected and analyzed 208 ex-vivo biopsy cores from PCa patients after prostatectomy (Table 1 and Supplementary Fig. 1A) using targeted bisulfite sequencing (68 − 9,848 reads per core, median: 3,367). The targeted DNA strand sequenced covers approximately 150 bp of the ONECUT2 gene-body in which a total of eight CpG sites are represented, including the two specific CpG sites (cg10835584, cg24771804) that correlate with expression (Fig. 3F). We aggregated the DNA methylation data based on the percentage of 0, 1, 2, 3, 4, 5, 6, 7, or 8 methylated site(s) on each DNA strand or read without considering the location within the region (Fig. 4A). After excluding pre-surgically treated cores, we analyzed 140 non-cancerous and 68 PCa lesions based on needle biopsy pathological reports (Supplementary Fig. 1B). In addition, in order to confirm the correlation between ONECUT2 gene-body methylation and mRNA expression in needle biopsy, we also extracted total RNA from each sample and performed multiplex qRT-PCR.

Heatmap representation of unsupervised clustering analysis revealed distinct DNA methylation patterns in adjacent-normal prostate and PCa lesions with statistically significant cancer-specific DNA hypermethylation (*p* < 1.9E-16 and 1.2E-15, respectively) in 7–8 CpGs in the *ONECUT2* locus (Fig. 4B). Therefore, these data can distinguish needle biopsies between adjacent-normal (benign or pre-malignant) and tumor cells from the patient samples (Supplemental Fig. 4). Furthermore, regarding the correlation of gene-body methylation and expression of ONECUT2, after converting the raw data of expression and DNA methylation to a Yeo-Johnson transformation, we performed a robust linear regression analysis, which revealed a significant positive correlation between ONECUT2 gene-body methylation levels and relative expression levels (R² value: 0.424) (Fig. 4C), which supported previous findings either in clinic database or cell lines by treatment of DNMTi (Figs. 1 and 2, and 3).

**Fig. 5:**
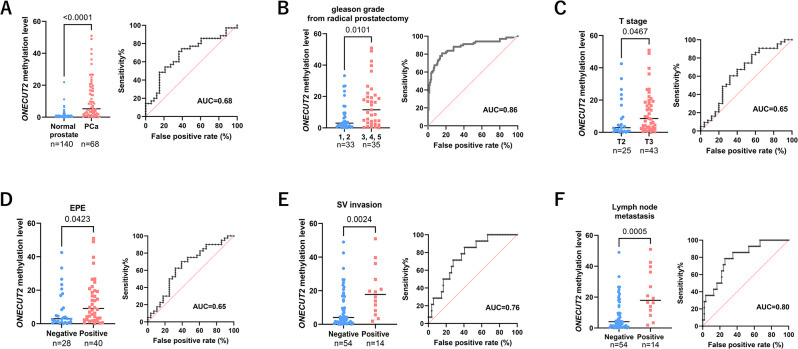
ONECUT2 gene-body DNA methylation and correlation with prostate cancer aggressiveness and AUC values in needle biopsies. (**A**) ONECUT2 gene-body DNA methylation based on the percentage of 7 or 8 methylated CpG sites and Receiver Operating Curve (ROC) analysis comparing normal prostate versus PCa. AUC values are indicated. (**B**) Gleason grade 1, 2 versus 3, 4, 5 (**C**) T2 versus T3 (**D**) Extra-prostatic extension (EPE) - negative versus positive (**E**) Seminal vesicle (SV) invasion - negative versus positive (**F**) Lymph node metastasis - negative versus positive

By focusing on these highly methylated strands (7–8 CpG sites), we found statistically significant ONECUT2 gene-body DNA hypermethylation in PCa lesions compared to adjacent-normal (benign or premalignant) prostate tissues (*p* < 0.0001 and AUC = 0.86) (Fig. 5A). These results also clearly demonstrated that fully methylated DNA strands are better to distinguish adjacent- normal and tumor cells than single CpG sites (*p* < 2.0E-10) or less than 7 CpG sites (Fig. 4B). Since the Illumina DNA methylation BeadArray, which is widely used for clinical specimens, is also dependent on a single CpG site, targeting fully or near fully methylated strands may provide improved specificity and sensitivity for a diagnostic biomarker.

Among PCa lesions, we explored the association between ONECUT2 gene-body DNA methylation levels and clinicopathological data. ONECUT2 DNA methylation was statistically significantly associated with indicators of disease aggressiveness, such as high Gleason grade (*p* = 0.0101 and AUC = 0.68) (3, 4, 5), T3, extraprostatic extension (EPE) positivity (*p* = 0.0423 and AUC = 0.65), particularly for seminal vesicle (SV) invasion positivity (*p* = 0.0024 and AUC = 0.76), and positive lymph node metastasis (*p* = 0.0005 and AUC = 0.80) (Fig. 5B-F). Furthermore, we investigated the association between ONECUT2 gene-body DNA methylation levels and PCa patient prognosis, revealing no significant association with PSA-based recurrence (Supplementary Fig. 5). These results suggest that pre-operative ONECUT2 gene-body DNA methylation may serve as a valuable biomarker for predicting not only its expression status but also aggressive PCa features.

## Discussion

ONECUT2 has been identified as a survival factor and a driver for tumor aggressiveness in CRPC and NEPC. ONECUT2 expression in prostate adenocarcinoma synergizes with hypoxia to suppress androgen signaling and induce neuroendocrine plasticity. A recent study has shown that the REST transcriptional complex, which is involved in neuroendocrine differentiation [39], directly binds to the ONECUT2 promoter and represses its expression. Another study reported that *RB1* loss, not *TP53* loss, induced ONECUT2 expression in CRPC [40]. Furthermore, a recent study has shown that ONECUT2 is not restricted to CRPC or NEPC but can function as a lineage plasticity driver in PCa [5]. This suggests that dysregulation of ONECUT2 may reflect not only an aggressive molecular phenotype but also potential for lineage plasticity, underscoring its relevance as a diagnostic biomarker for risk stratification. However, how ONECUT2 expression progressively increases from early to late PCa stage remains to be determined.

We uncovered a strong correlation between increased ONECUT2 expression and gene-body DNA hypermethylation, both of which are closely associated with aggressive PCa features including CRPC and NEPC. Elevated ONECUT2 expression and gene-body DNA hypermethylation emerged as predictive factors for favorable PSA relapse-free survival following prostatectomy. Recent findings showcasing the efficacy of the ONECUT2 inhibitor CSRM617 in suppressing tumor growth and metastasis in mouse models have positioned ONECUT2 as a potential therapeutic target [4]. Notably, the treatment response to CSRM617 exhibited a direct correlation with ONECUT2 expression in prostate cancer cell lines. These results underscore the potential of ONECUT2 expression as a predictive biomarker and a determinant for CSRM617 treatment selection. However, the lack of a well-established tool for measuring ONECUT2 expression in clinical specimens, such as in needle biopsies, has been a limitation. Here, we demonstrated a robust positive correlation between ONECUT2 gene-body DNA methylation and gene expression across several public databases, which we also confirmed this correlation not only in prostate cancer cell lines but also the key clinic specimen, needle biopsy before the surgery. Our ability to quantitatively analyze the DNA methylation levels of individual CpG sites in the ONECUT2 gene-body using targeted bisulfite sequencing and a modified COBRA assay positions ONECUT2 gene-body DNA methylation as a promising biomarker in prostate needle biopsies. These methods facilitate the monitoring of *ONECUT2* gene-body DNA methylation and expression before surgery, during treatment or to monitor disease progression. In addition, these results also clearly demonstrate that measuring DNA methylation of multiple CpGs in the same DNA strand is better to distinguish adjacent-normal and tumor tissues than interrogating individual CpG sites. Technologies that measure individual CpG DNA methylation, such as the Illumina Infinium DNA methylation BeadArrays, are commonly used for clinical specimens.

Given that aberrant cytosine methylation is a hallmark of human cancers and that DNA methylation is a stable molecular feature detectable in tissues, blood, and other bodily fluids, our study suggests that ONECUT2 gene-body DNA methylation status may serve as a valuable tool for predicting aggressive PCa features. This approach aligns with the broader trend of utilizing DNA methylation patterns as diagnostic and prognostic indicators in cancer research and clinical applications [8, 41]. Furthermore, the feasibility of measuring cell-free ONECUT2 gene-body DNA methylation in plasma from CRPC patients, as demonstrated in another study, underscores the potential clinical applicability of this biomarker [42]. Future studies will focus on analyzing the correlation between circulating (serum/plasma) and tissue-based ONECUT2 gene-body DNA methylation at clinically relevant time points, with the goal of establishing its utility as a minimally invasive biomarker for risk stratification and disease monitoring in PCa.

The inhibition of ONECUT2 expression through loss of DNA methylation via DAC treatment and the subsequent restoration of expression through remethylation in human PCa cell lines, highlights the potential direct regulatory role of gene-body methylation in ONECUT2 expression. Potential mechanisms underlying these processes have been elucidated in recent studies [14] and encompass regulation of alternative splicing [43], prevention of spurious transcription initiation [44], and stabilization of nucleosomes and transcriptional elongation [45]. Conversely, gene-body DNA hypomethylation has been demonstrated to disrupt these processes, leading to alterations in gene expression [13, 46, 47]. However, the present study does not definitively establish a direct causal mechanism by which gene-body DNA methylation regulates ONECUT2 expression; therefore, further mechanistic studies are required to clarify this relationship. Understanding the involvement of these processes in ONECUT2 regulation is crucial, given its essential role in the molecular biology of PCa. This knowledge holds significant implications for advancing our understanding of basic biological processes.

DNA methylation, a dynamic and pharmacologically reversible epigenetic mark, is an attractive therapeutic target in human cancers. The targeting alteration of DNA methylation in cancer cells, achieved through genetic ablation or pharmacological intervention, leads to profound inhibition of cancer cell growth [13, 46, 48–51]. DNA methylation inhibitors, such as decitabine (DAC), exhibit anti-neoplastic effects by reactivating tumor suppressor genes through promoter demethylation [41, 52] and down-regulating oncogenes through gene-body demethylation [13].

Despite numerous clinical trials assessing the efficacy of DNA methylation inhibitors in PCa treatment [53], none have gained FDA approval, potentially due to observed severe toxicities of these inhibitors at high doses [54]. A potential strategy to mitigate toxicity involves transient exposures to low doses of DNA methylation inhibitors that can still exert long-term anti-cancer effects by retaining DNA methyltransferase inhibition [55, 56]. This study contributes to this understanding by demonstrating that a low DAC dosage (0.3 µM) inhibits ONECUT2 expression in human PCa cell lines. Notably, preclinical research has shown that DAC (0.5 µM) restores sensitivity to enzalutamide in enzalutamide-resistant PCa cell lines [57]. Given the involvement of ONECUT2 in lineage plasticity driving neuroendocrine differentiation [40], the combination therapy of DAC and enzalutamide is a promising therapeutic strategy, especially by directly targeting ONECUT2 in PCa.

In this study, we established a positive correlation between ONECUT2 DNA methylation and gene expression, as evidenced in public databases that encompass CRPC and NEPC and our needle biopsy cohort. It is important to note that, while we did not directly analyze gene-body DNA methylation in needle biopsy samples specifically labeled as CRPC and NEPC due to a limited number of available clinical samples, our findings in this challenging context offer valuable insights. Obtaining CRPC and NEPC samples for DNA methylation analysis poses inherent difficulties, leading to a scarcity of such studies in the literature. Our study overcame some of these challenges by correlating ONECUT2 gene-body DNA hypermethylation with aggressive PCa features using prospectively collected needle biopsy samples. It should be noted that *ONECUT2* gene-body DNA methylation in our needle biopsy cohort was not associated with PSA recurrence-free survival. This may be attributed to factors such as a relatively short follow-up period or limitations in sample size. Nevertheless, our study contributes to addressing the complexities associated with DNA methylation analysis in the context of CRPC and NEPC. In addition, a recent study also demonstrates that ONECUT2 expression in hormone-naive tumors is more common than previously suggested [58]. All of these findings offer valuable insights into the aggressive features of prostate cancer.

## Conclusion

Our study demonstrates a strong association between ONECUT2 gene-body DNA methylation and aggressive prostate cancer features, validated using public Illumina Infinium methylation datasets as well as targeted bisulfite sequencing and qRT-PCR in our needle biopsy cohort. Given the availability of multiple approaches to assess DNA methylation [59], the consistent validation of our findings across different methods highlights the significance, robustness, and reproducibility of ONECUT2 gene-body DNA methylation in PCa. Our findings, supported by multiple analytical approaches, establish ONECUT2 gene-body DNA methylation as a promising biomarker prior to surgery, a potential tool for clinical decision-making, and a therapeutic target in PCa. These results underscore its clinical significance and lay the foundation for future translational research and therapeutic development.

## Supplementary Information

Below is the link to the electronic supplementary material.

Supplementary Material 1: Supplementary Figure 1: Needle biopsy study cohort (A) The schema of collecting needle biopsy samples from prostates after prostatectomy. (B) Sample selection flowchart. Supplementary Figure 2: ONECUT2 promoter DNA methylation status absent in prostate cells. (A) ONECUT2 DNA methylation in adjacent normal prostate and PCa tissues from TCGA PRAD. (B) ONECUT2 DNA methylation in normal prostate cell lines (RWPE1, PrEC) and prostate cancer cell lines (LNCaP, C4-2, C4-2B. 22Rv-1. VCaP, PC3, DU145, NCI-660). Supplementary Figure 3: The DNA methylation status of cg10835584 in the ONECUT2 gene-body in PCa. (A) The beta value of cg10835584 based on TCGA PRAD) (B) The beta value of cg10835584 in normal prostate, localized PCa,and metastatic PCa from GSE73549, GSE157272, and GSE174613 datasets. (C) Kaplan-Meier plots of PSA recurrence-free survival of PCa patients stratified by the beta value of cg10835584 after prostatectomy based on TCGA (PRAD), GSE83917, and GSE127985 datasets. (D) The relationship between ONECUT2 expression and the DNA methylation level of cg10835584 based on TCGA PRAD, E-MTAB6131, GSE83917, GSE107298, GSE183015 databases. Supplementary Figure 4: The detailed targeted sequencing data is based on percentage of methylated CpG site(s) in four needle biopsies from patient-14948 and patient-20142. Supplementary Figure 5: ONECUT2 gene-body DNA methylation levels in needle biopsies (cancer) based on PSA recurrence (negative or positive). Robust linear regression analysis comparing the relationship between ONECUT2 gene-body DNA mean methylation levels and mRNA relative expression compared to LNCaP in needle biopsies. These data were converted from raw data using a Yeo-Johnson transformation.

## Data Availability

All data from this study are included in the Methods section or Supplementary Information. Other data can be obtained from the corresponding author upon reasonable request.

## Abbreviations

PCa: Prostate cancer
DNMTi: DNA methyltransferase inhibitor
NEPC: Neuroendocrine prostate cancer
TCGA: Cancer Genome Atlas
PRAD: Prostate adenocarcinoma
GEO: Gene Expression Omnibus
RP: Radical prostatectomy
H&E: Hematoxylin and Eosin
mCOBRA: Modified combined bisulfite restriction analysis
CRPC: Castration-resistant prostate cancer
SWARM: Simplified Whole-panel Amplification Reaction Method
EPE: Extraprostatic extension
SV: Seminal vesicle
RT-PCR: Reverse Transcription-Polymerase Chain Reaction
qRT-PCR: quantitative RT-PCR
