# Supplementary material for: Gene-body DNA methylation of ONECUT2 predicts its expression and prostate cancer aggressiveness in needle biopsies
Source: Biomark Res. 2026 Jan 16;14:18. doi: 10.1186/s40364-026-00890-7 (PMC12829032; doi:10.1186/s40364-026-00890-7)
Supplement: Supplementary file 1 — Supplementary Material 1: Supplementary Figure 1: Needle biopsy study cohort (A) The schema of collecting needle biopsy samples from prostates after prostatectomy. (B) Sample selection flowchart. Supplementary Figure 2: ONECUT2 promoter DNA methylation status absent in prostate cells. (A) ONECUT2 DNA methylation in adjacent normal prostate and PCa tissues from TCGA PRAD. (B) ONECUT2 DNA methylation in normal prostate cell lines (RWPE1, PrEC) and prostate cancer cell lines (LNCaP, C4-2, C4-2B. 22Rv-1. VCaP, PC3, DU145, NCI-660). Supplementary Figure 3: The DNA methylation status of cg10835584 in the ONECUT2 gene-body in PCa. (A) The beta value of cg10835584 based on TCGA PRAD) (B) The beta value of cg10835584 in normal prostate, localized PCa,and metastatic PCa from GSE73549, GSE157272, and GSE174613 datasets. (C) Kaplan-Meier plots of PSA recurrence-free survival of PCa patients stratified by the beta value of cg10835584 after prostatectomy based on TCGA (PRAD), GSE83917, and GSE127985 datasets. (D) The relationship between ONECUT2 expression and the DNA methylation level of cg10835584 based on TCGA PRAD, E-MTAB6131, GSE83917, GSE107298, GSE183015 databases. Supplementary Figure 4: The detailed targeted sequencing data is based on percentage of methylated CpG site(s) in four needle biopsies from patient-14948 and patient-20142. Supplementary Figure 5: ONECUT2 gene-body DNA methylation levels in needle biopsies (cancer) based on PSA recurrence (negative or positive). Robust linear regression analysis comparing the relationship between ONECUT2 gene-body DNA mean methylation levels and mRNA relative expression compared to LNCaP in needle biopsies. These data were converted from raw data using a Yeo-Johnson transformation. [file 40364_2026_890_MOESM1_ESM.pdf]

Supplementary Figure 1

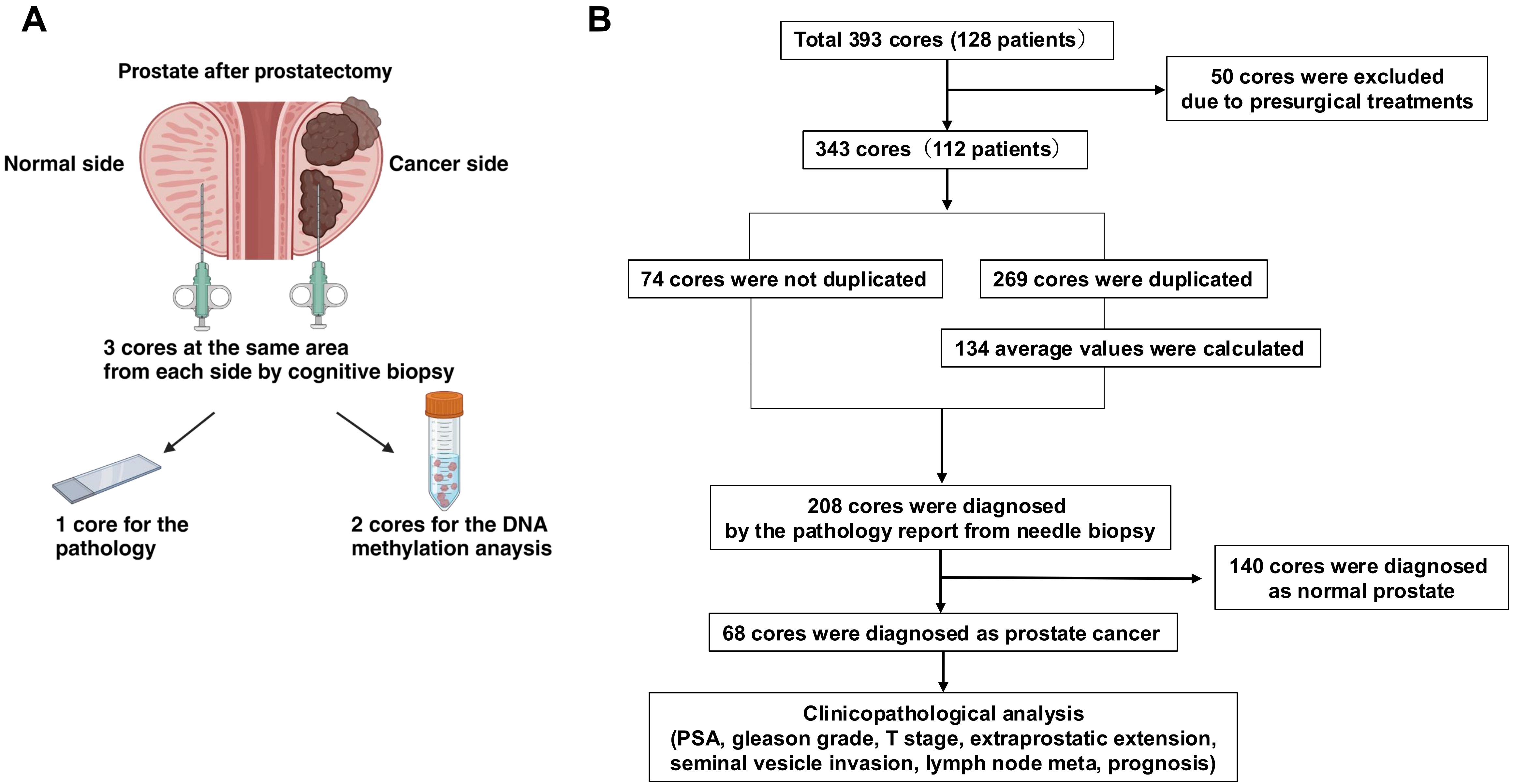

Supplementary Figure 2

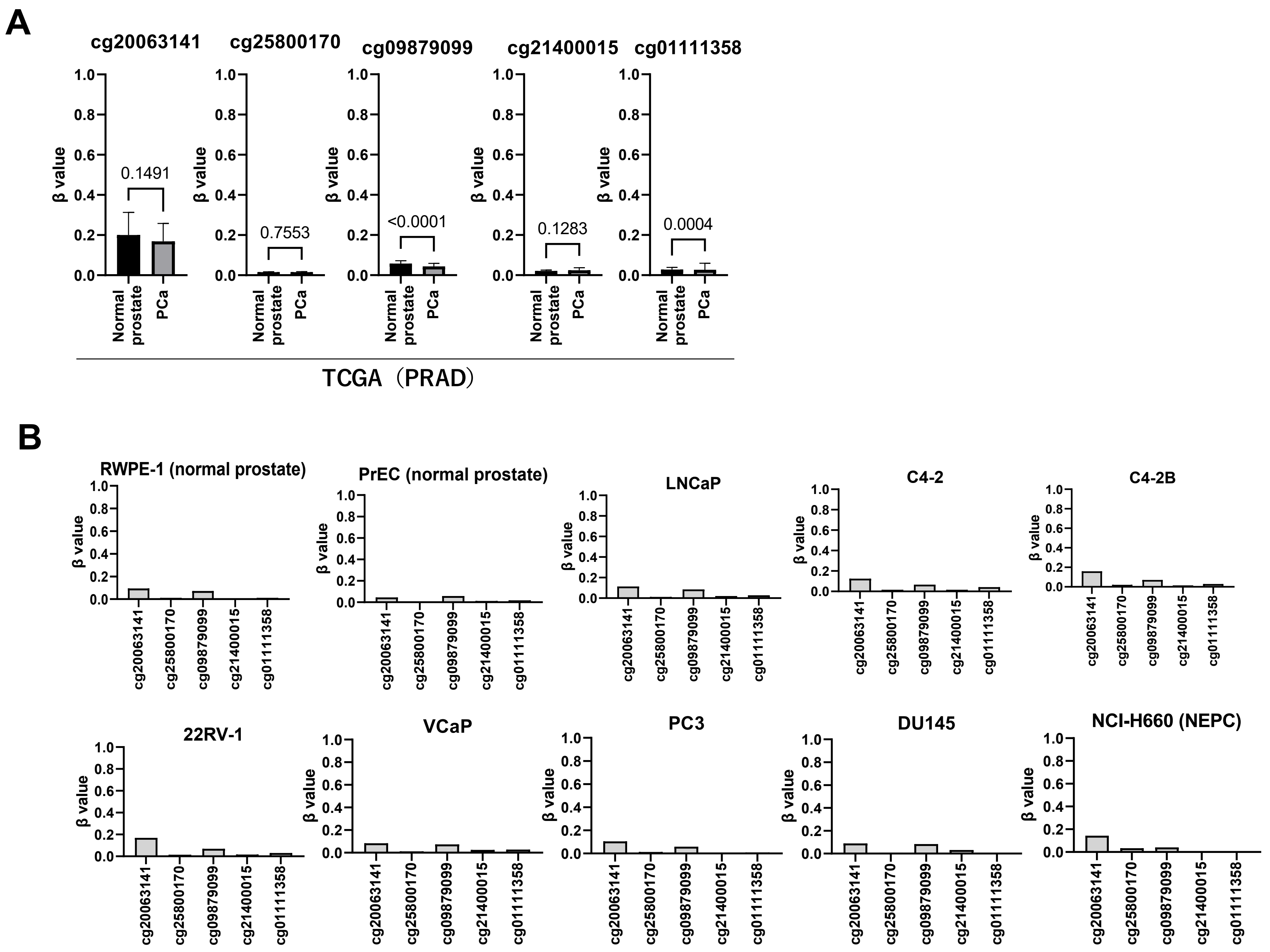

Supplementary Figure 3

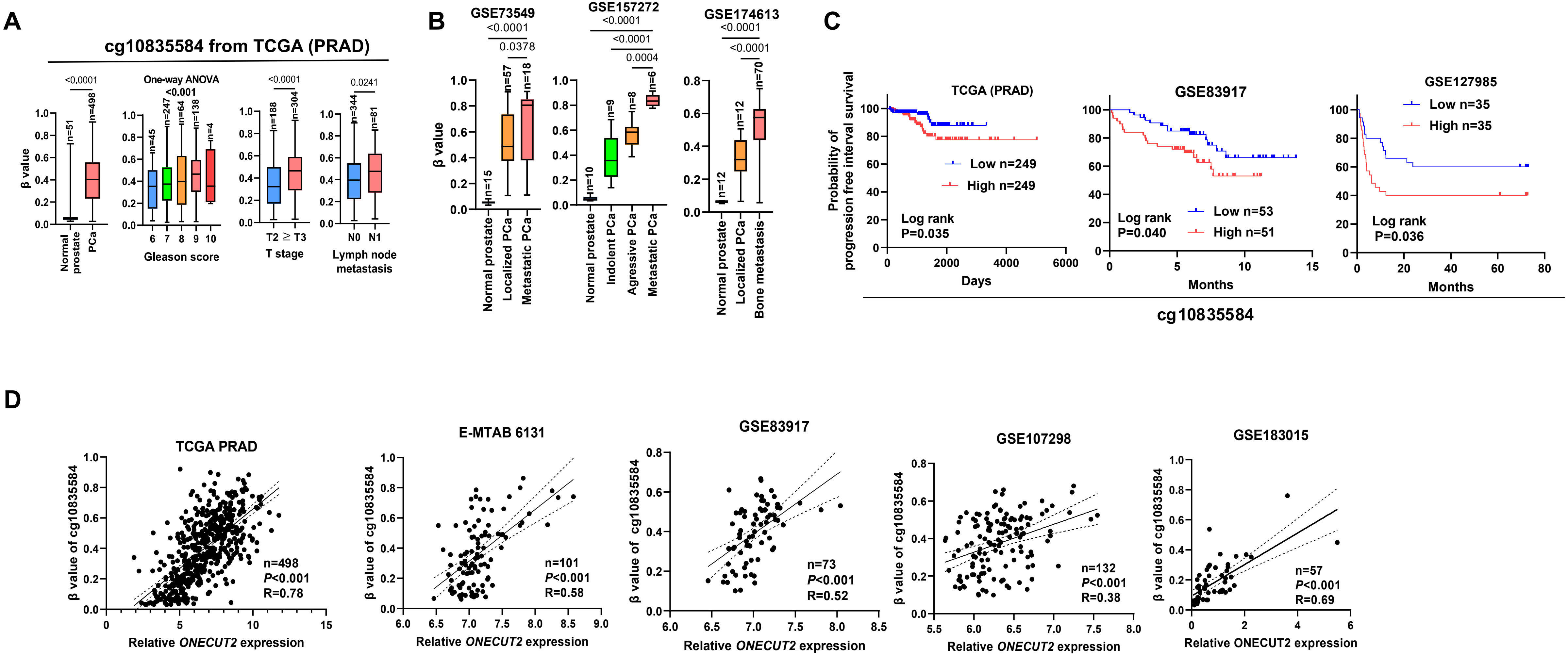

Supplementary Figure 4

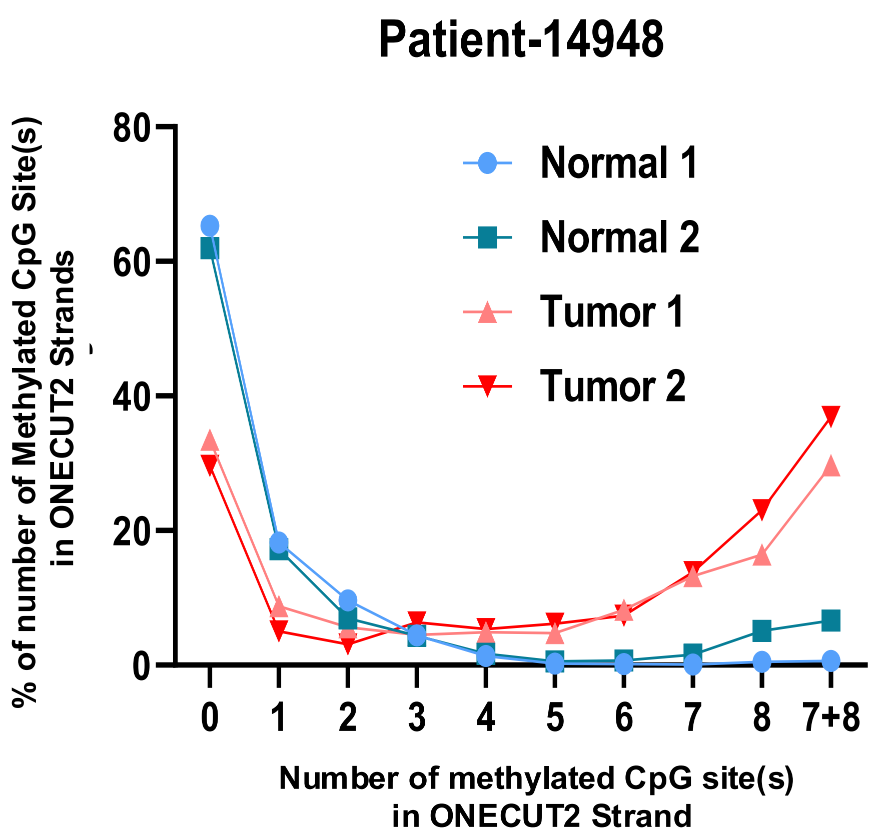

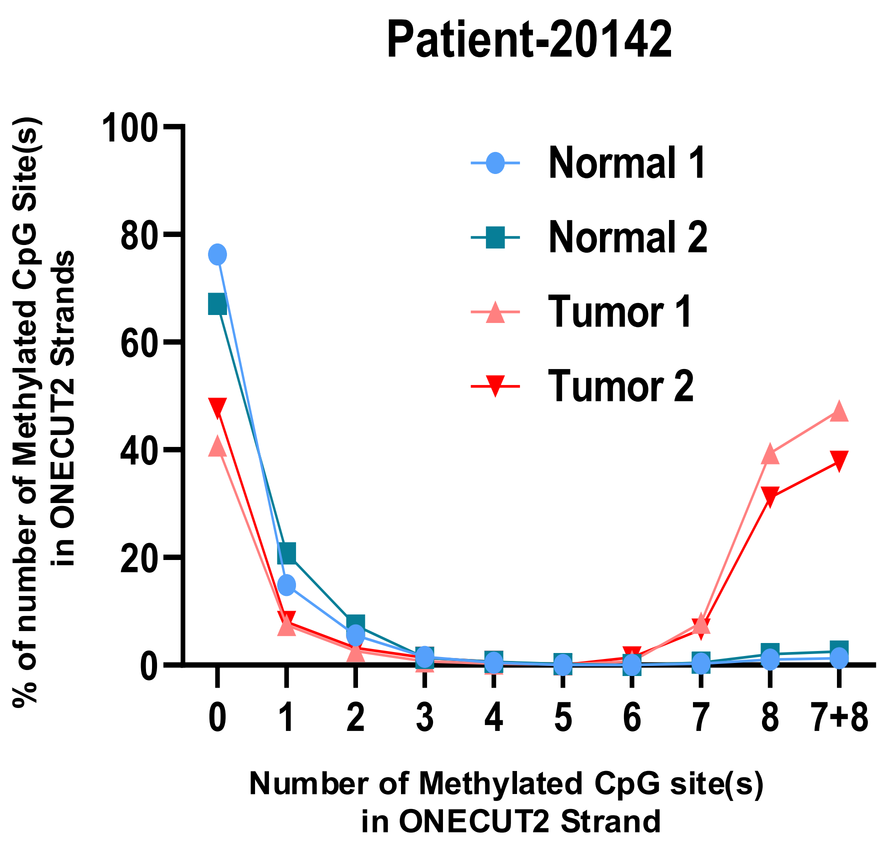

Supplementary Figure 5

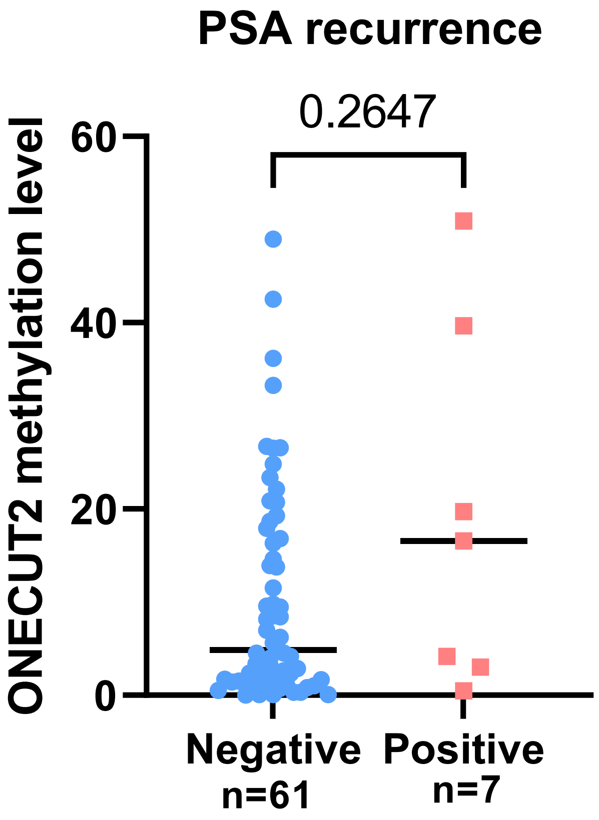

Supplementary Table 1 Primer sequences

Name	Primer sequences
ONECUT2 forward (qRT-PCR)	GAAGCGCTACAGTATCCCC
ONECUT2 reverse (qRT-PCR)	GTTTGCACGCTGCCAGG
ACTB forward (qRT-PCR)	CACCATTGGCAATGAGCGGTTC
ACTB reverse (qRT-PCR)	AGGTCTTTGCGGATGTCCACGT
ONECUT2 forward (multiplex qRT-PCR)	GATGTGGAAGTGGCTTCAGGA
ONECUT2 reverse (multiplex qRT-PCR)	TTTGCGTTTGCACGCTGCC
ONECUT2 probe (multiplex qRT-PCR)	TTCCAGCGCATGTCCGCCTTA
HPRT1 forward (multiplex qRT-PCR)	TGGACTAATTATGGACAGGACTGA
HPRT1 reverse (multiplex qRT-PCR)	AGCACACAGAGGGCTACAAT
HPRT1 probe (multiplex qRT-PCR)	TGTGATGAAGGAGATGGGAGGC
cg10835584 forward (mCOBRA)	GGGGTTTTTTGTTTTTTGTATTTTTTTTT
cg10835584 reverse (mCOBRA)	CAACGAATACACCTACGTAAAAAATCTA
cg24771804 forward (mCOBRA)	GTTGGGTTTTAGAGATTTTTAAATTAAGTAT
cg24771804 reverse (mCOBRA)	ATACCCCTAACAAAAACCACTA

Supplementary Table 2 CpG sites of ONECUT2

CpG	Group	Relation To Island
cg20063141	TSS1500 (promoter)	N_Shore
cg25800170	TSS1500 (promoter)	N_Shore
cg09879099	TSS1500 (promoter)	N_Shore
cg21400015	TSS1500 (promoter)	Island
cg01111358	TSS1500 (promoter)	Island
cg15892115	1st Exon	N_Shore
cg06275813	1st Exon	N_Shore
cg02250594	1st Exon	Island
cg20324206	1st Exon	Island
cg25547580	1st Exon	Island
cg23757446	1st Exon	Island
cg02455094	1st Exon	Island
cg16340618	Body (intron)	Island
cg12980128	Body (intron)	Island
cg07364220	Body (intron)	Island
cg12350762	Body (intron)	Island
cg06067372	Body (intron)	Island
cg01193769	Body (intron)	Island
cg03636532	Body (intron)	Island
**cg24771804**	Body (intron)	Island
cg00196827	Body (intron)	Island
cg04115544	Body (intron)	Island
cg20956738	Body (intron)	Island
**cg10835584**	Body (intron)	Island
cg16941302	Body (intron)	S_Shore
cg11817589	Body (intron)	S_Shelf
cg15276629	Body (intron)	Open Sea
cg07024339	2nd Exon	Open Sea
cg05480377	2nd Exon	Open Sea

Supplementary Table 2 CpG sites of ONECUT2

CpG	Group	Relation To Island
cg20063141	TSS1500 (promoter)	N_Shore
cg25800170	TSS1500 (promoter)	N_Shore
cg09879099	TSS1500 (promoter)	N_Shore
cg21400015	TSS1500 (promoter)	Island
cg01111358	TSS1500 (promoter)	Island
cg15892115	1st Exon	N_Shore
cg06275813	1st Exon	N_Shore
cg02250594	1st Exon	Island
cg20324206	1st Exon	Island
cg25547580	1st Exon	Island
cg23757446	1st Exon	Island
cg02455094	1st Exon	Island
cg16340618	Body (intron)	Island
cg12980128	Body (intron)	Island
cg07364220	Body (intron)	Island
cg12350762	Body (intron)	Island
cg06067372	Body (intron)	Island
cg01193769	Body (intron)	Island
cg03636532	Body (intron)	Island
**cg24771804**	Body (intron)	Island
cg00196827	Body (intron)	Island
cg04115544	Body (intron)	Island
cg20956738	Body (intron)	Island
**cg10835584**	Body (intron)	Island
cg16941302	Body (intron)	S_Shore
cg11817589	Body (intron)	S_Shelf
cg15276629	Body (intron)	Open Sea
cg07024339	2nd Exon	Open Sea
cg05480377	2nd Exon	Open Sea
